# Supplementary material for: Association between oral microbiome and seven types of cancers in East Asian population: a two-sample Mendelian randomization analysis
Source: Front Mol Biosci. 2023 Nov 21;10:1327893. doi: 10.3389/fmolb.2023.1327893 (PMC10702768; doi:10.3389/fmolb.2023.1327893)
Supplement: Supplementary file 1 [file DataSheet1.ZIP › Supplementary Materials/MR plots for tongue/tongue═╝/Colorectal cancer/pheno.272_to_colorectal cancer_forest.pdf]

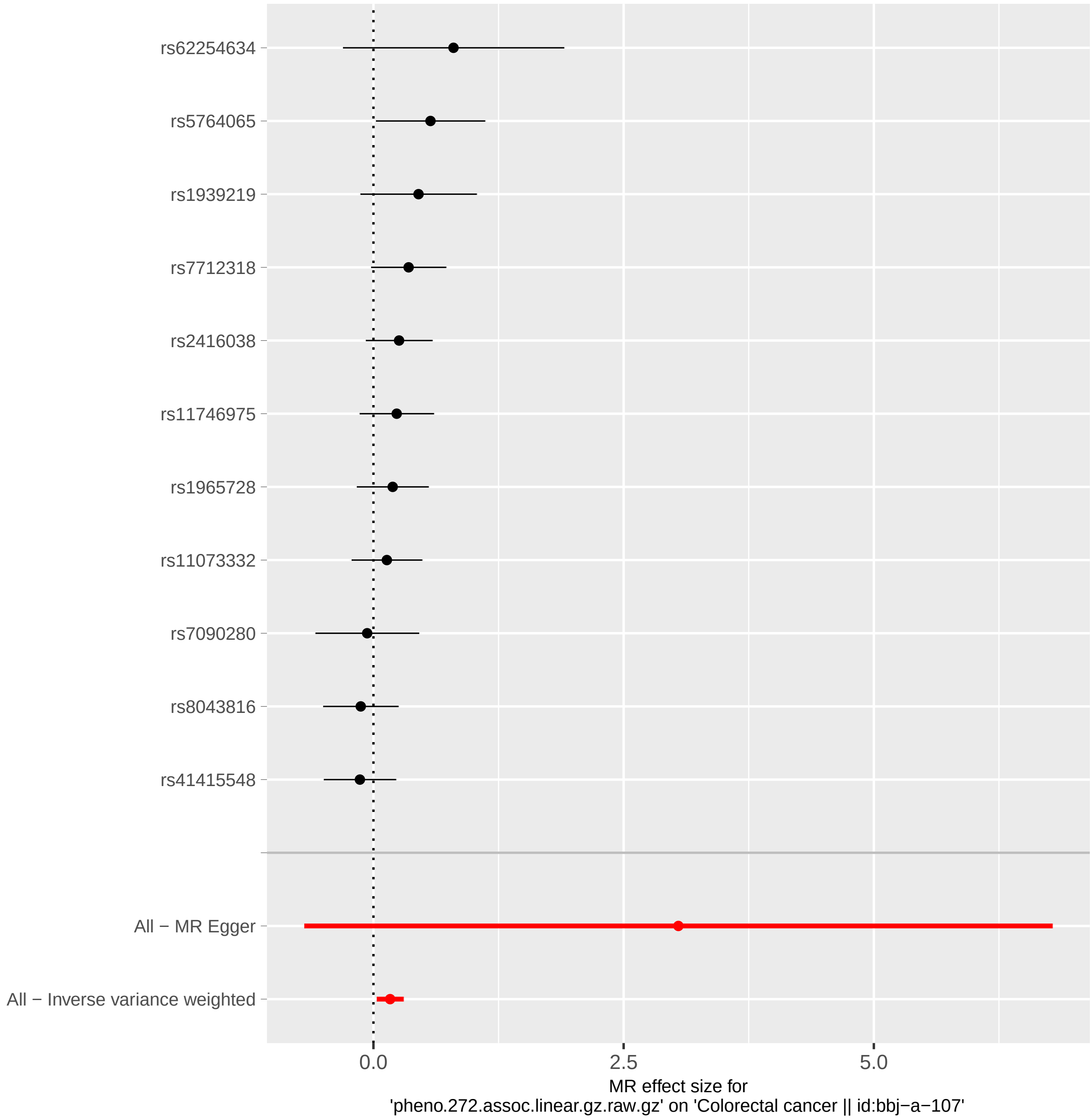

rs62254634
rs5764065
rs1939219
rs7712318
rs2416038
rs11746975
rs1965728
rs11073332
rs7090280
rs8043816
rs41415548
All − MR Egger
All − Inverse variance weighted
0.0
2.5
5.0
MR effect size for
'pheno.272.assoc.linear.gz.raw.gz' on 'Colorectal cancer || id:bbj−a−107'
